# Evidence for compositionality in baboons *(Papio papio)* through the test case of negation

**DOI:** 10.1038/s41598-022-21143-1

**Published:** 2022-11-10

**Authors:** Isabelle Dautriche, Brian Buccola, Melissa Berthet, Joel Fagot, Emmanuel Chemla

**Affiliations:** 1 Laboratoire de Psychologie Cognitive, Aix-Marseille University, CNRS, Marseille, France; 2 Department of Linguistics, Languages, and Cultures, Michigan State University, East Lansing, MI USA; 3 Institut Jean Nicod, Département d’Etudes Cognitives, Ecole Normale Supérieure, PSL University, EHESS, CNRS, Paris, France; 4 Laboratoire de Sciences Cognitives et Psycholinguistique, Département d’Etudes Cognitives, Ecole Normale Supérieure, PSL University, EHESS, CNRS, Paris, France

**Keywords:** Animal behaviour, Human behaviour, Evolution of language

## Abstract

Can non-human animals combine abstract representations much like humans do with language? In particular, can they entertain a compositional representation such as ‘not blue’? Across two experiments, we demonstrate that baboons (*Papio papio*) show a capacity for compositionality. Experiment 1 showed that baboons can entertain negative, compositional, representations: they can learn to associate a cue with iconically related referents (e.g., a blue patch referring to all blue objects), but also to the complement set associated with it (e.g., a blue patch referring to all non-blue objects). Strikingly, Experiment 2 showed that baboons not only learn to associate a cue with iconically related referents, but can learn to associate complex cues (composed of the same cue and an additional visual element) with the complement object set. Thus, they can learn an operation, instantiated by this additional visual element, that can be compositionally combined with previously learned cues. These results significantly reduce any claim that would make the manipulation and combination of abstract representations a solely human privilege.

## Introduction

A basic mental operation that underlies the structural complexity of language is compositionality^1^, which states that the meaning of an expression is determined by the meaning of its constituents and the rules used to combine them. Compositionality is hypothesized to be a defining feature of human language and unique to human thought^2–4^, but its origin in evolution is controversial.

A rich body of field and lab research shows that animals can combine signals into sequences^5–13^. For instance, putty-nosed monkeys combine two different referential calls signaling predators to trigger a group movement^14^, enculturated apes are able to spontaneously produce several referential signals sequentially^5^, and chimpanzees may even combine facial expressions and gestures in a productive way^13^. Yet, in all cases and more generally across this literature, there is no clear evidence that the structure of these combinations adjusts the meaning of what is being communicated by the individual signals in a systematic manner and, thus, that they qualify as cases of semantic compositionality^9,13,15–17^.

Here we investigate abstract compositional representation in non-human primates through the study of negation, one of the most basic yet fundamental functional operations that exemplify compositionality. Negation is typically evidenced in language, where it can take various forms cross-linguistically: French has the words *non* and *pas*, Japanese the suffix *-nai*, English the prefix *un-*. Such negations may be seen as assuming a large range of semantic roles. For example, the negative particle *no* in English can be used to express falsity, refusal (e.g., in response to an order), displeasure, disbelief, exasperation, and more^18^. One salient technical property is that negation is always the negation of something, and as such is a typical case of functional application (the application of a function to an argument), the basic form of compositionality. In doing so, negation entails a relation where a mental representation is obtained by contrast to another source mental representation: truth is defined in contrast to falsity and vice-versa, pleasure to displeasure and vice-versa, a set (e.g., triangles) to its complement (non-triangles). We will focus on the latter case of set complementation, as it contains all the relevant pieces that make negation interesting and powerful: a compositional manipulation of abstract representations, without it being restricted to a narrow domain (truth/falsity).

Various forms of negation have been successfully taught to non-human animals: rejection, refusal, and other concepts that have negation-like functions^19^. As for set complementation, two types of studies are relevant, but not decisive.

First, animals have been shown to comply with ‘mutual exclusivity’, i.e., inferring that a new label is not associated with objects for which they already know a name^20,21^ or inferring that a food reward is located in a location B when the only other possible location A is empty^22,23^. However, these types of studies are not designed to show that a label is assigned to all other possible objects, nor do they show that animals’ behavior is based on the logical relation between the representation ‘absent’ and ‘present’ rather than holding separate representations for these two concepts. Critically, it has been argued that these studies concern proto-negation^19^, the operation that maps one concept to a complementary concept but only within a pre-established set of contrary pairs (e.g., absent/present), as opposed to a more general form of negation that may not require such previously established pairs (i.e., if a description applies to an arbitrary set of situations, its negation will apply to all the other situations; e.g., blue/not-blue). Such a form of negation has been argued to require a human-language-like system of representation^24^.

Second, animals succeed in “non-matching-to-sample tasks”: when presented with a cue, they can learn to select, in a target pair, the object that is not the cue^25,26^. However, these paradigms do not necessarily come with a control condition for learning concepts that are of similar complexity but not based on negation. Nor do they address the more “linguistic” side of the equation, i.e., trying to trigger such negative behavior from the stimuli only, rather than at the level of the task (see Experiment 2).

We present two experiments testing whether baboons (*Papio papio*) display a capacity for compositionality focusing on the critical case of negation. Compositionality requires that the constituent units of an expression have independent meanings. We define meaning here as the association between a cue and a referent. Experiment 1 tested whether baboons can learn negative, compositional, meanings (such as ’not-blue’), and Experiment 2 tested whether they can associate a negative meaning with a visual cue.

## Experiment 1

We explored whether baboons are better at learning negation-like associations between a cue and a set of stimuli (e.g., a blue patch of color being the cue for non-blue objects) than arbitrary associations. Baboons ($n=24$) were tested in a matching-to-sample task using a testing procedure in which they had free access to computer-controlled operant conditioning setups with touch screens^27^.

**Figure 1:**
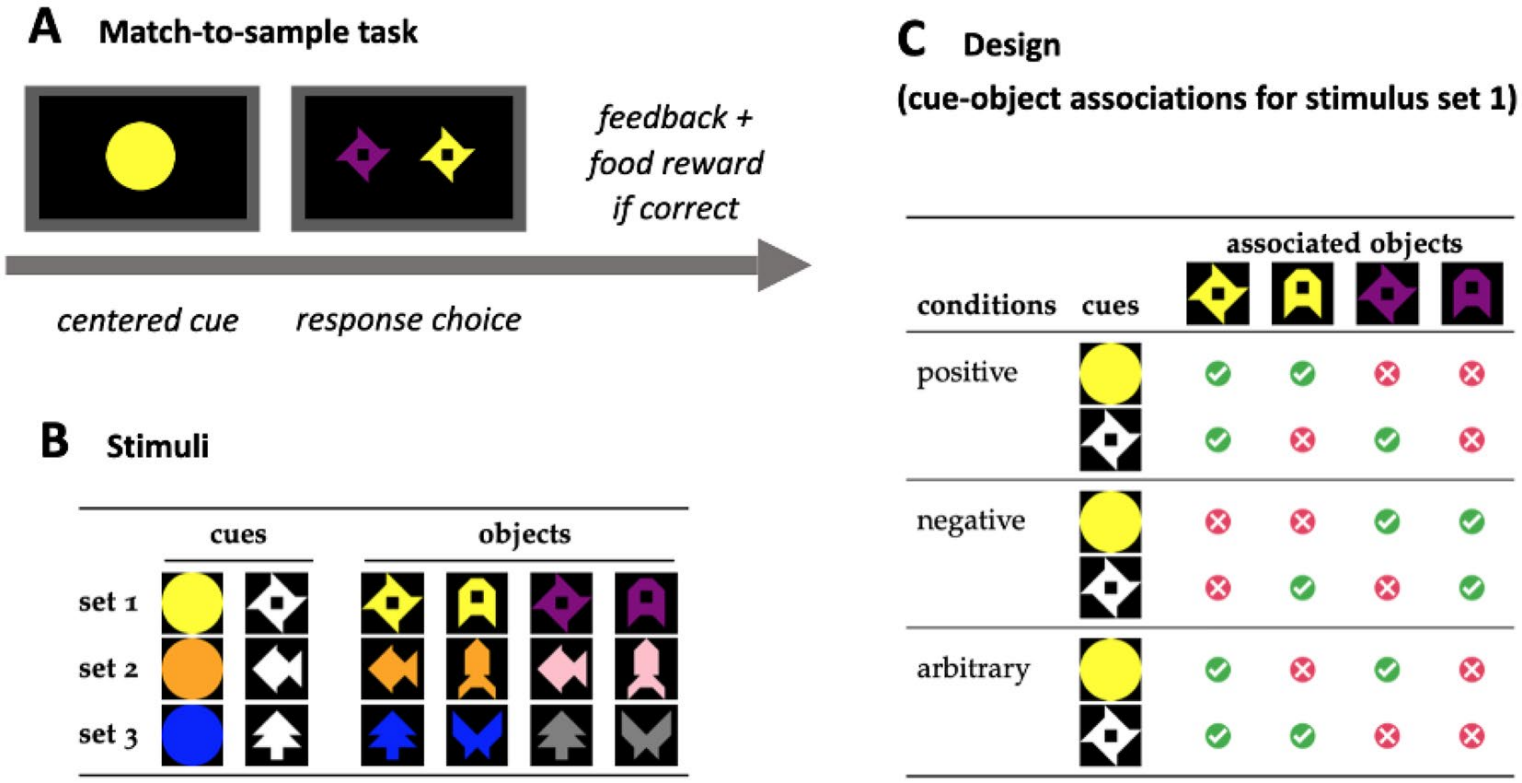
Task and experimental design (Experiment 1). (**A**) Baboons were taught cue-object associations in a matching-to-sample task that consisted of a series of trials in which a cue (e.g., a yellow circle) was presented centrally before baboons could choose which of two objects was associated with it. (**B**) In consecutive stages, baboons were exposed to different stimuli sets. (**C**) For each stimulus set, a baboon learned associations between cues and objects based on conditions (positive, negative, or arbitrary).

In each trial, baboons were first presented with one of two possible cues, either a color cue (a circle filled with a target color) or a shape cue (a target shape filled with white). After they touched the cue, two colored shapes were presented side by side on the screen, and the task was then to touch which of these two objects was associated with the cue to obtain a food reward (see Fig. 1). In the positive condition, the object-cue association was iconic: the cues (e.g., the yellow circle) shared one visual dimension with the objects triggering a reward (e.g., the yellow shuriken or the yellow letter A). In the negative condition, the responses were systematically the opposite, so that the cues (e.g., the yellow circle) were effectively associated with the complement set of iconically related objects (e.g., the purple shuriken and the purple letter A). In the arbitrary condition, the cues (e.g., the yellow circle) did not share any consistent visual dimension with their target responses (e.g., the yellow shuriken and the purple shuriken), yet as in the other conditions, the set target objects were coherent in some dimension (e.g., shape). For each condition, there were two target responses per cue, which, to avoid ambiguities, were never presented as the two proposed options.

If baboons readily assume that a cue is more likely to be associated with the object that the cue resembles the most, i.e. they show an *iconicity bias*, they should learn faster in the positive condition (although it is not always found that non-human animals and human infants display an iconicity bias (i.e.,^28–32^)). Our crucial prediction is that if baboons can represent negative concepts, therefore showing a capacity for compositionality, they will learn faster in the negative condition than in the arbitrary condition: despite the fact that all expected responses in the negative condition go opposite to an iconicity bias, participants will capitalize on the systematicity of the (negative) association.

### Method

#### Data availability

The data and the script for the analysis in this paper are available online: 10.17605/OSF.IO/VZ8UR.

#### Ethical standards

This research conformed to the Standard of the American Psychological Association’s Ethical Principles of Psychologist and Code of Conduct and received ethical approval from the ethics committee of the French Ministry of Education (approval APAFIS 2717-2015111708173794 v3). The study is reported in accordance with ARRIVE guidelines.

#### Participants and apparatus

The data provided by 24 Guinea baboons (*Papio papio*, 17 females; age range: 2–24 years) from the CNRS primate facility (Rousset-sur-Arc, France) were analyzed. The participants were tested using ten automatic computerized learning devices for monkeys^27^ that were freely accessible from the baboons’ living enclosures. Each test system comprised a touch screen, a food dispenser, and an automated radio frequency identification (RFID tag) of the participants. This allows us to test the individuals without capturing them, which improves animal welfare in experimental research^33^.

The experiment was made available to all 25 animals in our facility, who participated in the study on a voluntary basis. Of the 25 participants, 1 failed to complete the first condition they were assigned to (see ‘Procedure and learning criteria’ below). The experiment was stopped when at least 4 participants per group completed the 3 conditions (see ‘Task and conditions’ below).

#### Materials

There were three sets of stimuli (see Fig. 1). Each set of stimuli was made of four objects varying systematically along two binary dimensions: color and shape. The three stimuli sets differed in the pairs of shapes (shuriken/letter A, fish/rocket, tree/butterfly) and the pairs of colors (yellow/purple, orange/pink, blue/gray) they featured. All shapes were composed of the same set of basic elements (tangrams) and thus had the same total area.

For each stimulus set, we constructed a color cue and a shape cue. The color cue was of the color shared by two target objects (the other two objects were of a different color) shaped as a circle, a shape not used in any of the stimuli sets. The shape cue was of the shape shared by two target objects, colored in white, a color not used in any of the stimulus sets. Each image was created as a bitmap file with $250 \times 250$ pixels and presented on the screen as a square of 6 cm, corresponding to a visual angle of 11.4$^{\circ }$ at a distance of 30 cm.

#### Task and conditions

We used a matching-to-sample task: In each trial, participants were presented with a cue (a sample), after which they had to choose between two objects (comparison stimuli) the one associated with the cue. The goal of the task was to learn the associations between each cue and their corresponding objects in each stimuli set. Each cue was always associated with two target objects (out of 4 possible for a given stimuli set).

There were three conditions (see Fig. 1C). In the positive condition, the object-cue associations were iconic: the associated object shared one visual dimension with the cue (e.g., the yellow circle cue was associated with the two yellow objects of the image set). In the negative condition, the cues were associated with the complement set of iconically related objects (e.g., the yellow circle cue was associated with the two non-yellow, purple, objects of the image set). In the arbitrary condition, the form of the cue could not be linked to the set of objects it was associated with, but the coherence of the associated objects was preserved (e.g., the yellow circle cue was associated with the two shuriken shapes: the yellow shuriken and the purple shuriken).

The three conditions were implemented in a different order to three different groups of participants such that all conditions were presented first, second, or third across the three groups. The stimuli sets were presented in the same order to the different participants (so that they would be matched with different conditions across groups).

Trials could be of 3 different types depending on how the target object was iconically related to the cue. Some trials were positive, in the sense that the cue shared a dimension with the rewarded response and not with the distractor (like all trials in the positive condition, by construction of the cue; and 1/4 of the trials in the arbitrary condition); some trials were negative, the opposite (like all trials in the negative condition; and 1/4 of the trials in the arbitrary condition); and some trials were neutral, in the sense that the cue might have shared a dimension with both or none of the target objects.

#### Procedure and learning criteria

Once the participant entered a test booth, they were immediately recognized by the test system with their RFID tag. As shown in Fig. 1A, a trial started with the presentation of a cue centered in the middle of the screen. Once participants touched the cue picture, two comparison stimuli appeared on each side of the screen. Baboons learned the associations between the cue and one of the comparison stimuli through positive reinforcement: touching the correct stimulus (as defined by the condition) cleared the screen and delivered a food reward. Touching the incorrect stimulus triggered a 3-s timeout indicated by a green screen. Participants were allowed a maximum of 5 s to respond. Aborted trials were not recorded, and were presented again in the next trial. The inter-trial interval was set to 3 s.

Within each condition, items were presented as a succession of blocks of 16 trials randomized within blocks. These 16 trials cover all possible combinations of target-distractor pairs ($2 \times 2$; see Fig. 1C) for each cue (2 total) with the target appearing once on the right and once on the left (2).

A condition was considered learned when the participants made no more than three errors per block for two consecutive blocks (a general accuracy criterion), and no more than 1 error per triplets of cue and comparison stimuli (to ensure that participants’ errors are not systematic) or when participants reached a maximum of 4000 trials ($n = 3$ across all participants, all in the arbitrary condition). Once these criteria were reached, the experiment progressed to the next condition.

#### Inclusion criteria

Because participants have different levels of expertise with the matching-to-sample task that could exhibit significant outliers in learning time, we decided a priori to remove outliers using a repeated Grubbs test^34^ applied in each condition at the alpha level of 0.05. In total 4 condition blocks were removed, the negative and positive condition blocks for one participant, and the positive condition for two more participants. All other condition blocks for which the learning criteria were reached were included in the analysis. Including the excluded condition blocks in the analysis did not change the pattern of results. Of the 24 participants that completed at least one condition, 17 participants completed the 3 conditions (7 in the order arbitrary-positive-negative, 6 in the order negative-arbitrary-positive, and 4 in the order positive-negative-arbitrary), 3 completed 2 conditions, and 4 completed a single condition. This left us with 18 participants in the positive condition, 21 in the negative condition, and 22 in the arbitrary condition. Participants took on average 873 trials to reach the learning criterion across conditions ($\text {SE} = 127$; $\min = 64$; $\max = 4000$).

#### Analyses

##### Preliminary analysis (early iconicity bias)

To measure whether participants were more likely than chance to choose an iconic response, we calculated an iconicity index for each participant and each condition separately. We defined the iconicity index as the proportion of iconic responses in positive or in negative trials (i.e., in trials where an iconic response is possible) minus the chance level to choose an iconic response. In the arbitrary condition, the chance level was defined as participants’ accuracy in neutral trials to account for an individual learning performance. In the positive and the negative conditions, however, the chance level was 0.5 since there were no other trial types in these conditions that could be used to better estimate the chance level taking into account participants’ performance at the task. To give a concrete example, suppose that in the arbitrary condition the proportion of iconic responses is 0.6 in positive trials and 0.5 in negative trials. Participants’ performance in neutral trials is 0.55. The iconicity indexes for positive trials is thus $0.6 - 0.55 = 0.05$ and $0.5 - 0.55 = -0.05$ for negative trials. That is, the participant was more likely than chance to choose an iconic response on positive trials (iconicity index greater than 0) but less likely than chance to do so in negative trials (iconicity index less than 0). We modeled such an iconicity index using a mixed model specified as:

$$\begin{aligned} \texttt {iconicityIndex} \sim~{} \texttt {trialType + (1|participant)} \end{aligned}$$

using the lme4 package^35^ in R. Because the model was singular, we built on recent advances in Bayesian statistics (using the brms package in R^36^) to model our by-participant estimates^37^. We tested whether the intercept for each level of trialType (positive; negative) was significant. For each parameter, we report estimates (B), estimated error (EE), and the 95% credible interval (CI). If zero lies outside the credible interval, then we conclude there is sufficient evidence to suggest the estimate is different from zero.

##### Main analysis (compositionality effect)

To quantify the ease with which different types of associations (positive, negative, arbitrary) are learned, we modeled the number of blocks (16 trials) needed to reach the learning criterion using a mixed model specified as:

$$\begin{aligned} {\texttt {Nblock}} \sim \texttt {condition + (1|participant)} \end{aligned}$$

**Figure 2:**
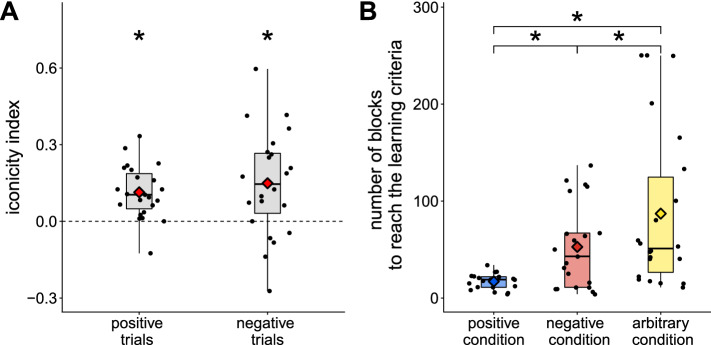
Results (Experiment 1). (**A**) Early iconicity bias. The iconicity index is the difference score between the proportion of iconic (positive) responses and participants’ expected performance (measured as the performance in neutral trials in the arbitrary condition where both or none of the target objects shared a dimension with the cue, and taken to be 50% in the positive and negative conditions), computed at the beginning of each condition (first 10% of trials to complete this condition). Each dot represents the measure for a participant in a given condition. Consistent with the presence of a bias towards iconic responses, baboons initially displayed an above-chance preference for iconic responses (positive iconicity index), regardless of the feedback they received (that is, for both positive and negative trials). Asterisks denote statistical significance ($>0$). (**B**) Global negation effect. Average number of blocks to reach the learning criterion in each condition (positive, negative, arbitrary). Diamonds show condition means, and each dot represents a participant. The positive condition was learned the fastest, and crucially the negative condition was learned faster than the arbitrary condition. Asterisks denote statistical significance.

As can be observed in Fig. 2B, the distribution of learning times (in number of blocks) was not normally distributed and followed a log-normal distribution. Because such a distribution is not supported by standard frequentist mixed model analysis methods, and because the number of observations per participant was not sufficient to produce robust participant-level estimates in the frequentist approach, we also used Bayesian statistics to fit a log-normal distribution to the data, using individual variation across participants to leverage our by-participant estimates.

### Results

#### Preliminary result: early iconicity bias

In the positive condition, all trials are iconic (the cue and target share a visual dimension). Our first analysis asked whether participants have an initial bias towards such iconic responses, and in fact irrespective of the global condition, i.e. of the actual cue-object associations being reinforced. For each participant and condition, we focused here on the first 10% of trials before successful completion of that condition by that participant ($\text {mean} = 94 \text { trials}$, $\text {SE} = 12$, $\min = 16$, $\max = 400$). As illustrated in Fig. 2A, we found that participants initially choose the iconic response at rates greater than chance in both positive trials, where the iconic response is the rewarded response ($B = 0.11$, $EE = 0.04$, $CI = [0.05, 0.17]$), and also in negative trials ($B = 0.15$, $EE = 0.03$, $CI = [0.08, 0.20]$), where the iconic response is not the rewarded response, thus resulting in below-chance accuracy in the negative condition during the initial blocks of trials.

#### Main result: compositionality effect

Critical to our question is the ease with which the different conditions (positive, negative, arbitrary) are learned. As shown in Fig. 2B, the positive condition ($M = 17$ blocks, $SE = 2$) was learned faster than the negative condition ($M = 53$ blocks; $SE = 10, B = 0.83, EE = 0.30, CI = [0.34, 1.32]$) and the arbitrary condition ($M = 87, SE = 18, B = 1.34, EE = 0.30, CI = [0.83, 1.84]$). Crucially, participants were faster to learn the negative condition than the arbitrary condition ($B = 0.51, EE = 0.29, CI = [0.04, 0.99]$), despite the initial disadvantage for the former due to the early iconicity bias.

Overall then, baboons have an early bias towards positive (iconic) responses, and the positive condition is eventually learned the fastest. This bias leads participants to initially perform below chance in negative trials, where the iconic response is not the target response. But crucially this is quickly overcome in the condition where all trials are negative, eventually leading participants to perform better in the negative condition than in the arbitrary condition.

### Discussion

Baboons were able to learn negative associations: they were able to quickly match a ‘yellow circle’ cue with non-yellow objects. These results are not due to “inhibition” or “reversal learning”, as in studies in which animals typically learn an association, and then later learn to not choose the matching category^38^. Unlike these studies, the negative condition was not preceded by a corresponding positive condition using the same cue-object association that would serve as the basis of reversal learning. To be sure, we observed no effect of order, to the point that the effects are visible even when we restrict the analysis to the first condition a participant would see (baboons learned faster in the negative condition than in the arbitrary condition, $B = 1.29, EE = 0.46, CI = [0.51, 2.05]$, see details in the online analysis script). Finally, the comparison with the arbitrary condition, in which the same set of responses were taught but with different cues, shows most directly that it is the (negative) association between cue and referents that helped learning.

There are, however, three alternative interpretations to our results. First, it could be that baboons were faster to learn in the negative condition compared to the arbitrary condition because they focused on the cue feature that is *not* iconic, i.e. not represented in the comparison stimuli. For instance, when presented with a yellow circle cue, they would ignore the color feature and concentrate on the shape feature, therefore conferring an advantage to the negative condition (where circle would be an arbitrary cue associated with non-yellow objects) compared to the arbitrary condition (where circle, a shape, would be in competition with the target object set, i.e. shuriken shapes). This would predict that there is no advantage for the positive condition over the negative condition (where circle would also be an arbitrary cue associated with yellow objects). This is, however, not what we find: the positive condition is learned the fastest, suggesting that baboons tend to focus on the cue feature that matches rather than the one that discriminates.

The second alternative interpretation of our results is that baboons learned faster in the negative condition compared to the arbitrary condition not as a result of our experimental manipulation but as a result of the extra penalties they receive in the negative condition. Indeed, because baboons start with an iconicity bias, they initially receive more negative feedback in the negative condition compared to the arbitrary condition. There are, however, two important points that make that interpretation unlikely. First, it is not always the case that negative feedback is more efficient than positive feedback for learning in baboons^39^, and second, even assuming it is, there is also more ground to cover in terms of learning in the negative condition compared to the arbitrary condition because of that initial iconicity bias.

The third alternative interpretation of our results is that baboons, instead of learning a negative representation (‘not yellow’), rather learned to associate a cue with a simple property. In our experiment there were only two possible colors occurring in a given condition, such that the negative concept ‘not yellow’ corresponded to the simple and “positive” category of purple objects. Hence participants in the negative condition could learn to associate the yellow cue with purple objects rather than with non-yellow objects. Importantly, however, the cue-object associations were similarly simple in the arbitrary condition: the yellow cue was also associated with a single property (all shuriken objects), and this condition was harder to learn than the negative condition. This suggests that it is the negative correspondence between cue and category that helped learning. Yet, it remains possible that the yellow cue made the color dimension salient, that is, prompted participants to pay more attention to colors, such that participants were faster to learn in the negative condition (where the target objects share the same color) compared to the arbitrary condition (where the target objects share the same shape). Thus, Experiment 2 addresses this issue, by asking whether baboons’ response to a complex stimulus reflects the responses to its component parts, rather than holistically responding to the combination as a single, distinct meaningful unit, that is, whether they demonstrate a capacity for compositionality.

## Experiment 2

We explored whether baboons can associate a negation meaning with a cue, thereby showing an ability to accommodate a functional meaning for a visual cue and to integrate this logical meaning in a compositional manner. We used a task similar to the one described above.

**Figure 3:**
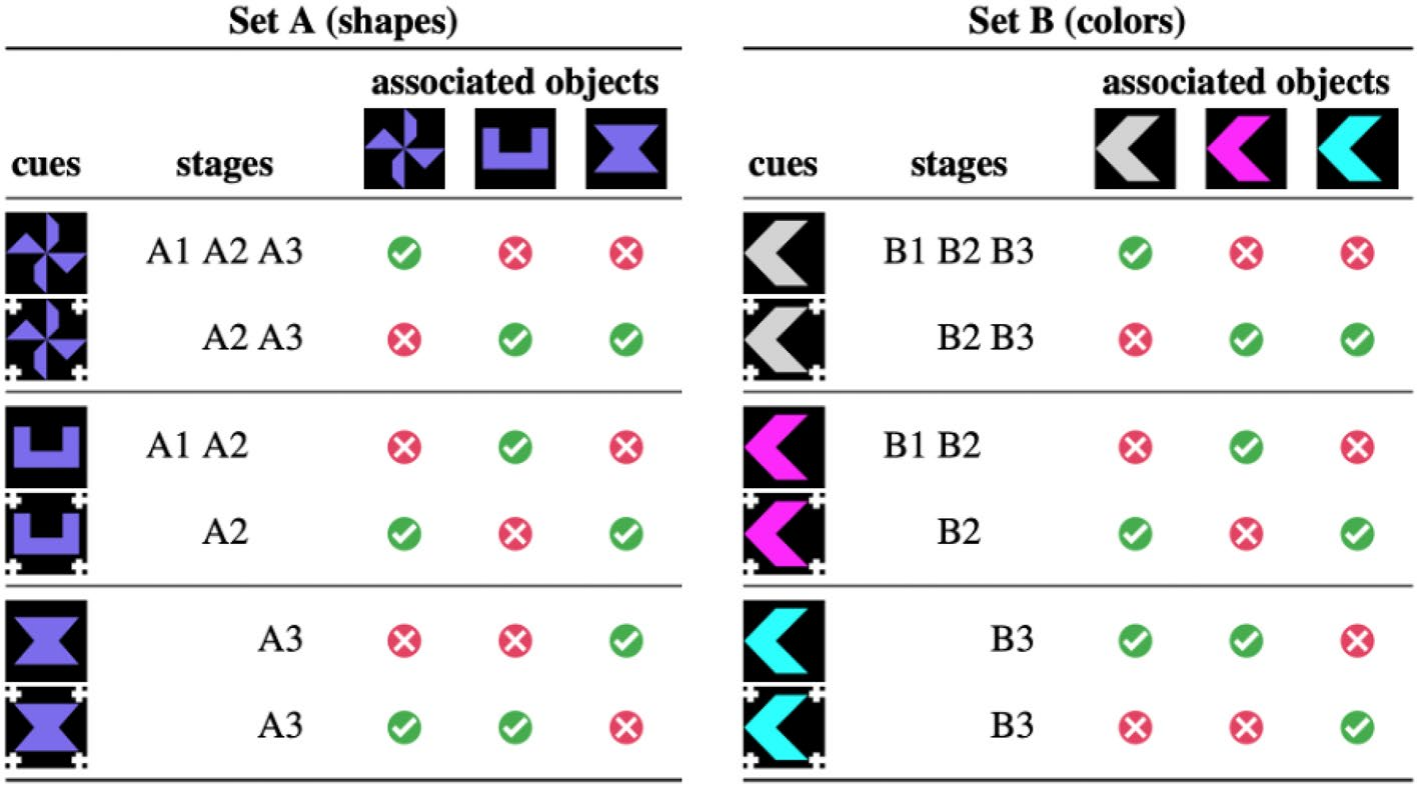
Experimental design (Experiment 2). In each trial, a cue (in rows) was presented, followed by two objects, one object triggering a reward for that cue (checkmark) and one not. Each cue was introduced at different stages. Baboons went through two sets of stimuli based on shapes and then colors (A then B). In each case, baboons first learned associations between atomic cues and the object that is the same as the cue (stages A1/B1). Complex cues were introduced at stages A2/B2. They were made of the same atomic cues complemented by 4 white crosses, and their reward was associated with the objects that do not resemble the embedded atomic cues. In A3, participants learned a generalization of these associations applied to a new pair of atomic and complex cues, and in B3 baboons were asked to learn a reversed association for such a new pair (compare the last two lines on both sides).

Baboons ($n = 6$) learned in the following different stages (see also Fig. 3). At stage A1, they learned to associate atomic cues (a U shape and a wind turbine) with an object that is the same as the cue (similar to the positive condition of Experiment 1). At stage A2, the baboons were presented with the same atomic cues, and with the atomic cues augmented with a “negation morpheme” (complex cues, e.g., a U shape surrounded by 4 crosses) and for which the reward was reversed (i.e., effectively making the crosses a negation marker). At stage A3, baboons were exposed to a block similar to A2, with atomic and complex cues, but implemented with a pair of new atomic and complex cues.

During a second set of stages, baboons went through a similar phase, but objects varied not by shape but by color. They went through stages B1 (cue/responses variation) and B2 (negation) entirely analogous to A’s. Crucially, at stage B3 when a new type of cue was introduced (i.e., a new color), the meanings of atomic cues and complex cues were reversed. If, through all the stages before, participants learned the meaning of atomic cues and learned how meaning is systematically modified by the negation morpheme (i.e., show a capacity for compositionality), they should find the last stage B3, in which the negation regularity is violated, harder than its mirror image A3.

### Method

#### Data availability

The pre-registration, the data, and the script for the analysis in this paper are available online 10.17605/OSF.IO/C43GW.

#### Participants

6 Guinea baboons (*Papio papio*, 4 females; age range: 4–11 years) participated in the study. An additional 18 animals participated in the study but were not included in the analysis because either they failed to complete the experiment ($n = 6$), or they were part of a second group of participants ($n = 12$) for whom the stages were presented in reverse order (stages B followed by stages A; see Task and conditions) that underwent a technical issue (no phase (i) was presented, see Task and conditions below, and there were implementation errors in the learning criteria). Note that despite this technical error, the final sample size remains comparable to other primate cognition studies^40^. The experiment was stopped when at least 6 participants completed all the stages. Participants were tested on the same apparatus as Experiment 1.

#### Materials

There were two sets of three object stimuli. In the first set, the three objects had a different shape (a U-shape, a sand glass, a wind turbine; all blue) and in the second set, a different color (gray, pink, cyan; all arrows). For each stimulus set, there were three atomic cues that were identical to the objects they were matched with, and three complex cues that were composed of the atomic cues complemented with a “negation morpheme”: four white crosses placed at each corner of the stimulus picture.

#### Task and conditions

We used a matching-to-sample task as in Experiment 1. Each atomic cue was associated with the object it was identical to, and each complex cue to the two other objects in the set (i.e., the objects that were not identical to the atomic cue it was composed of).

Baboons learned incrementally through two sets of stages, A and B. Stage A used the first set of stimuli and stage B, the second. The stages were as described previously (see Fig. 3), with some finer structure for the first stages of each set of stimulus (A1 or B1). There, baboons learned to associate two atomic cues with an object that was the same as the cue, and this was done in two substages (i) and (ii). In substage (i), the atomic cue (e.g., either the wind turbine or the U-shape) was always followed by the same two comparison stimuli (e.g., a wind turbine and a U-shape). To pass this substage, baboons could not rely on the comparison stimuli only (they were always the same two, up to order) and had to refer back to the cue that was presented earlier. At substage (ii), another type of trial was added in which a third object, never associated with a cue (e.g., the sand glass), was sometimes part of the two response options. These trials were introduced only at a second substage because they can be passed without paying attention to the cue (“never click on the sand class”), so avoiding them would ensure that baboons would learn the matching-to-sample task and pay attention to the cue. We also tested a second group of participants, which was supposed to go through the two blocks A and B in the opposite order, but did not quite do so for technical reasons. Full details are available in the online repository.

#### Procedure and learning criteria

The procedure was the same as in Experiment 1. In each stage, trials were presented as a succession of blocks of 8 (for A1 and B1) or 16 trials (A2, B2, A3 and B3). For all stages but A1a and B1a, these trials corresponded to all the target-distractor combinations possible at each stage for each cue counterbalanced for right and left positions. For A1a and B1a, the total number of such trials was 4, but was doubled to match programming constraints.

A stage was considered learned when participants made no more than 6 errors on the last 32 trials (corresponding to 4 blocks of trials in stages A1 and B1, with a maximum of 2 errors per block, and 2 blocks in the other stages, with a maximum of 3 errors per block), and no more than 25% of errors per triplets of cue and comparison stimuli. On the final A3 stage and B3 stage, we added a stopping criterion when participants reached a maximum of 4,000 trials ($n = 3$, all in the B3 stage). The experiment stopped when participants reached a maximum of 15,000 trials across all the stages.

#### Inclusion criteria

We included all participants that finished the experiment, as our crucial test relied on the comparison between A3 and B3 (the last stage). As in Experiment 1, we planned to remove outliers with a Grubb test while keeping a minimum of 12 participants in our sample. Since 6 participants finished the experiment, no outliers were removed.

#### Analyses

##### Preliminary analysis (early iconicity bias; not pre-registered)

Similarly to experiment 1, we measured how likely participants were to choose the iconic target when presented with a new pair of atomic and complex cues in stages A3 and B3. The iconicity index was defined as the proportion of iconic responses minus the chance level of choosing such a response (0.5) for each participant and each cue type (atomic, complex). For instance, if the proportion of iconic responses is 0.6 across trials where the complex cue is presented, the iconicity index would be $0.6 - 0.5 = 0.1$ which is positive and thus indicates that participants where more likely to choose the iconic response by chance in these trials. As in experiment 1, we modeled the iconicity index using a mixed model specified as:

$$\begin{aligned} \texttt {iconicityIndex} \sim \texttt{cueType + condition + (1 | participant)} \end{aligned}$$

Following Experiment 1 we present the Bayesian statistics in the results section.

##### Main result (compositionality effect)

If baboons can assign a meaning to the negative morpheme we introduced (the four crosses), we expected they would take more time to learn B3 (which violates the previously learned meaning rules) than A3. To quantify the ease of learning in A3 and B3, we modeled the number of blocks needed to reach the learning criterion using a mixed model specified as:

$$\begin{aligned} \texttt {Nblock} \sim \texttt{condition + (1|participant)} \end{aligned}$$

Since the sample size was too small to appreciate the form of the distribution of learning times, we present the results of a model fitting a normal distribution to the data (the results are qualitatively the same using a log-normal distribution or a frequentist approach).

Other planned analyses concerned the comparison between groups (and thus could not be run because of the absence of the group of participants due to technical issues) and can be found in the online analysis script. We note that all results from the group which contained technical errors were in the predicted direction (the negation blocks were learned and the anti-negation block was the hardest; all information that is available in the online repository).

### Results

#### Preliminary result (early iconicity bias)

**Figure 4:**
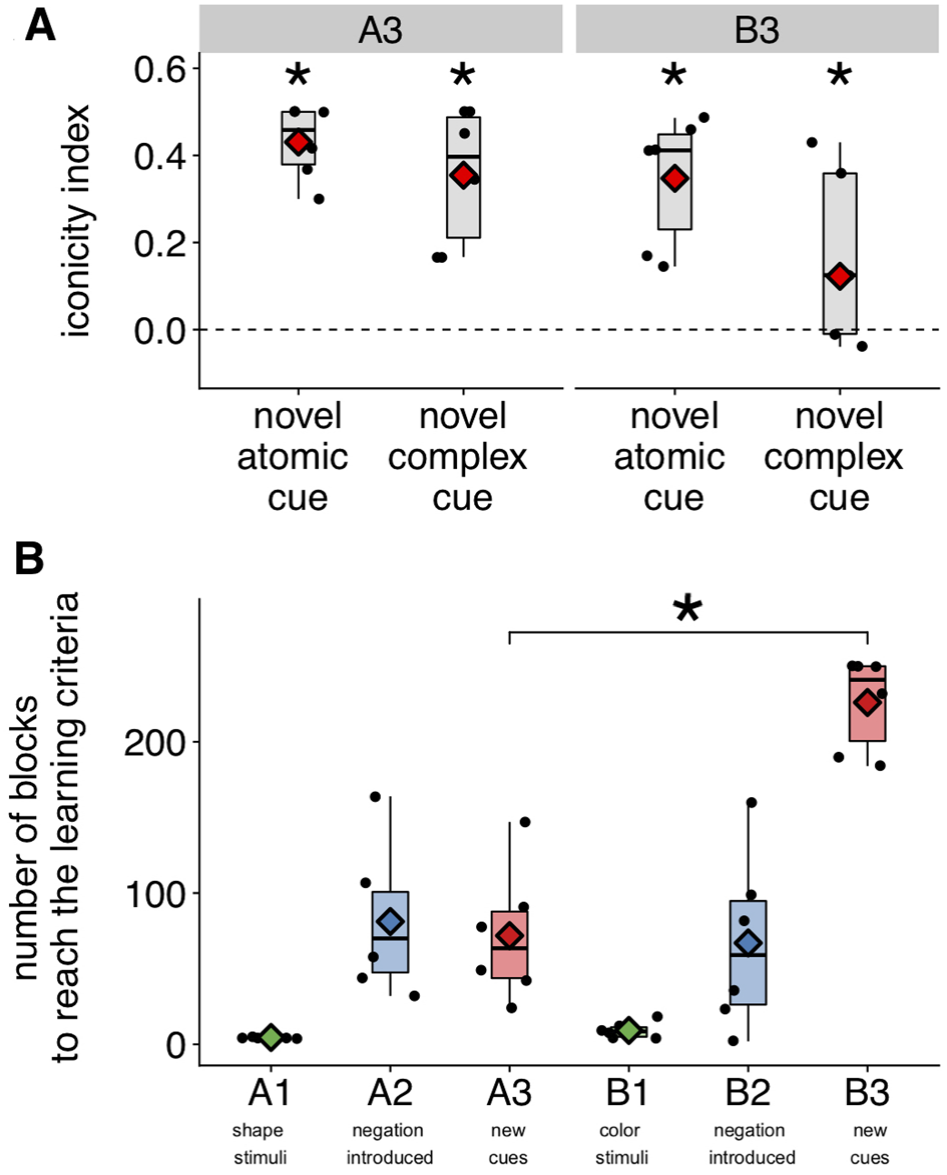
Results (Experiment 2). (**A**) Early iconicity bias. The iconicity index is the difference score between the proportion of iconic responses and the chance level, i.e. 0.5., computed at the beginning of the critical learning stages A3 and B3 (first 10% of trials) for the novel atomic cue and the novel complex cue. Each dot represents a participant. Consistent with Experiment 1, baboons initially displayed an iconicity bias: they chose the iconic responses above chance, regardless of the feedback they received (that is, for both novel atomic and complex cues in A3 and B3). Asterisks denote statistical significance ($>0$). (**B**) Compositionality effect. Average number of blocks needed to reach the learning criterion in each learning stage. A3 was learned faster than B3. Diamonds show condition means. Each dot represents a participant. Asterisks denote statistical significance.

Experiment 1 uncovered an “iconicity bias” whereby participants tend to choose the object that resembles the cue independently of the feedback received. We tested how this bias affects learning novel cues in stages A3 and B3, where both the novel atomic cue and its paired complex counterpart (composed of the atomic cue and the negative element) share a visual dimension with one of the objects. As in Experiment 1, we focused here on the first 10% of trials before successful learning of A3 and B3 for each participant (A3: mean = 115 trials, SE = 30, min = 32, max = 240; B3: mean = 360 trials, SE = 21, min = 288, max = 400) and computed such an index for the novel atomic cue and the novel complex cue separately. As shown in Fig. 4A, in A3, participants initially chose the iconic responses at a rate greater than chance for both the atomic cue, where the iconic response is the rewarded response ($B = 0.38$, $EE = 0.08$, $CI = [0.22, 0.55]$) and the complex cue, where the iconic response is not the rewarded response ($B = 0.24$, $EE = 0.10$, $CI = [0.06, 0.43]$). In B3, where this time the iconic response is the rewarded response for the complex cue but not for the atomic cue, participants also chose the iconic responses at a rate greater than chance, although this was only significant for the atomic cue (atomic cue: $B = 0.35$, $EE = 0.08$, $CI = [0.19, 0.53]$; complex cue: $B = 0.13$, $EE = 0.09$, $CI = [-0.05, 0.30]$). Participants chose the iconic responses at a greater rate when presented with atomic cues than when presented with complex cues ($B = 0.20$, $EE = 0.01$, $CI = [0.18, 0.23]$), suggesting that the iconic bias is stronger for atomic cues (because it is identical to the iconic response) and/or that it is mitigated by participants’ previous learning experience (as they learned that complex cues are associated with non-iconic responses).

#### Main result (compositionality effect)

Critical to our question is the time taken to learn the conditions A3 and B3. As can be seen in Fig. 4, baboons quickly reach the learning criterion in all conditions except in the final stage B3, when they take 3 times as many blocks (which is significantly more than for the counterpart A3; $M_{A3} = 72$ blocks, $SE_{A3} = 18$, $M_{B3} = 226$ blocks, $SE_{B3} = 13$, $B = 153.51$, $EE = 20.3$, $CI = [120.9, 188]$). Importantly, B2 was learned as fast as A2 ($M_{A2} = 81$ blocks, $SE_{A2} = 20$; $M_{B2} = 67$ blocks, $SE_{B2} = 24$; $B = -13.68$; $EE = 19.74$; $CI = [-44.25, 19.96]$), suggesting that the difference between A3 and B3 is not the result of stages in B being harder to learn (either because of the stimuli set used in B or because of fatigue accumulating throughout the experiment).

In sum, during the critical learning stages A3 and B3, baboons start with an initial iconicity bias to associate both cue types, atomic and complex, with the stimuli that resemble the cue the most. This bias leads participants to initially perform below chance for the novel complex cue in A3 and for the novel atomic cue in B3, both associated with non-iconic responses. Critically, however, this is quickly overcome in A3, where the novel cue-object associations are a generalization of the mappings learned in the previous learning stages, but not in B3, where the novel cue-object associations violate such mappings.

### Discussion

Across several learning stages, baboons learned to associate atomic cues with objects that are the same as the cues, and to associate complex cues (composed of the same atomic cue and an additional visual element) with the complement object set. Our results show that baboons were faster to learn a generalization of these associations applied to a new pair of atomic and complex cues (A3) than when they were asked to learn a reversed association for such a new pair (B3). This suggests that baboons may effectively have learned to associate the negation “morpheme” with some negative function and apply that function to the embedded atomic cue. Failure to generalize this rule in B3 would thus lead to greater learning time.

There may be an alternative, low-level, interpretation of these results that does not call upon any compositional ability. When presented with an atomic cue or a complex cue in A3 and B3, participants are biased to choose the iconic response (see Fig. 4A). This could be because participants ignore the negative morpheme when presented with complex cues or because they are subject to an iconicity bias as long as the cue bears some resemblance to one of the possible responses. However, complex cues could lead to less iconic responses when participants occasionally pay attention to the negative morpheme by chance: these small numbers of correct trials could help participants to learn that the complex cue in A3 is not associated with the iconic response. On the contrary in B3, it would be harder for participants to learn that the atomic cue is associated with the non-iconic response because there is no visual element in the cue that would help them to shed their iconicity bias. While we cannot entirely dismiss this possibility, our data provide little support for this alternative interpretation since participants are also tested on another, already learned, pair of atomic and complex cues in stage A3 and B3 and respond with high accuracy in these trials (80% accuracy for both cues in the first 50 blocks of trials), suggesting that they are not ignoring the negative morpheme.

One may worry that generalization of the negation rule is not visible elsewhere, as both A3 and B2 could be easier to learn than A2 if participants capitalized on what they have learned in previous stages. Critically though, A2 is not directly comparable to A3 and B2. In A2, they learned two complex cues while in A3, they learned a novel pair of atomic and complex cues. Regarding A2 vs. B2, while this time participants learn two complex cues in these phases, A2 uses shape as the relevant matching dimension while B2 uses color. Because in both cases we cannot be sure which associations (two complex cues vs. one pair of complex and atomic cues) or which stimulus feature (shape vs. color) are easier to learn, the critical generalization test remains between stages A3 and B3 which are both directly comparable since participants are learning the same type of associations and since learning in these stages is not impacted by stimulus feature, or presentation order (as performance in A2 and B2 is comparable).

We conclude that the most likely interpretation of our results is that participants, while subject to an early iconicity bias, learned to associate a visual element with some type of negative function (see “General discussion”) and apply this function to the embedded atomic cue. That is, they can compose cognitive representations. Generalizing this rule in A3 helped them to depart faster from their initial iconicity bias to learn the novel pair of atomic/complex cues, while failure of this generalization in B3 put them on the wrong track.

## General discussion

Compositionality is one of the key building blocks of human language and human thought, allowing individuals to create or derive the meaning of complex expressions from the meaning of their constituents parts and the rules used to combine them. To date, there is no evidence that non-human animals are able to compose cognitive representations. In two experiments, we investigated baboons’ ability to learn set complementation, a prime case study for exploring the origin of the compositional capacity across our phylogeny. We found that baboons can learn an operation that can be compositionally applied to previously learned representations. In the following, we discuss how the present set of results advance our understanding of iconicity, negation, and compositionality.

### Iconicity

Iconic signals resemble their referent, and thus can be used to communicate a variety of meanings on the spot in the absence of pre-existing, arbitrary conventions. The ability to readily understand those naturally referential signals have been assumed to play a central role in the acquisition and the emergence of language (e.g.,^41,42^). Yet, while human adults readily understand iconic signals, this capacity is not always observed in children or non-human animals^28–32^. In particular, great apes fail to readily identify the referent of an iconic gesture or acoustic signal^19,30,32^. In the present set of experiments, we observed that baboons were biased towards choosing the object that shared a visual property with the cue regardless of the feedback received (as in the negative trials of Experiment 1 or as in step 3 of Experiment 2). Here, it was methodologically useful as it made the precondition of learning to-be-negated words easy (iconicity speeds up learning by a factor 5 in Experiment 1). Yet, it suggests that baboons display a spontaneous sensitivity to iconic correspondences in simpler, non-linguistic cases, involving only the visual modality. Further studies should explore whether such an iconicity bias can be found in a wider range of dimensions, beyond color and shape, which the present baboons have extensive experience with.

### Negation

Negation has never been found in otherwise complex animal communication systems^43,44^, nor is there much evidence that any part of animal communication systems contains “functional” elements like negation, conjunction, or tense. This does not mean that non-human animals cannot perform such abstract operations. Some studies have shown an ability of animals for negative-like reasoning (e.g.,^22,23^). Yet, as outlined in the introduction these results fall short of demonstrating that non-human animals can represent set complementation. In particular, they fail to show that (1) non-human animals represent ‘not-A’ as all the possible elements that are not A, instead of associating ‘not-A’ with a single concept, B, and that (2) non-human animals hold a logical relation between a concept and its complement. While Experiment 1 has the same shortcomings (see discussion of Experiment 1), in Experiment 2, baboons learned to associate the negative morpheme with a function and apply that function to a previously learned representation. Is that function really set complementation? We showed that baboons could learn that ‘not-A’ applies to B and C, thus moving beyond studies using pairs of contrary concepts. While our current experiment was not designed to this end, further study could be designed to test how baboons generalize to never before seen stimuli in this task. An alternative possibility to set complementation is that the negative morpheme could just mean “different from” rather than “not”, but importantly this would still be a negative function in the broad sense (or proto-negation^19^). Yet critically, we showed that baboons learned the logical connection between the ‘negative’ morpheme and its attached cue. This suggests that they can learn a logical operation that bears some of the properties of negation.

### Compositionality

Finally, our main result concerns compositionality. Negation is a very clear signal of compositionality because it completely reverses the meaning of the elements it applies too. In that sense, semantic compositionality has never been found previously in animal communicative systems nor has it been evidenced in enculturated great apes^9,15–17^. In Experiment 2, baboons learned to associate atomic cues with objects that are the same as the cues, and to associate complex cues (composed of the same atomic cue and an additional visual element) with the complement object set. Baboons were faster to learn a generalization of these associations than when they were asked to learn a reversed association. This suggests that they learned the meaning of atomic cues and learned how meaning is systematically modified by the negation morpheme rather than holistically learning each complex cue as a single meaningful unit. In the future, one could try to show that baboons immediately and spontaneously understand new complex cues in a compositional manner, although one-shot learning of this type is hard to measure, as it may be masked by noisy experimental responses and independent biases (such as the strong iconicity bias present here) (see also^45^ for additional evidence that stronger effects can be observed during learning than during generalization in matching-to-sample tasks in baboons). What our results do suggest is that the ability to combine abstract representations is not unique to humans.

### Conclusion

The ability to combine abstract representations underpins many of our psychological competences: the acquisition and understanding of natural language, logical inferences, concept learning, reasoning, propositional attitudes, and so on. Evidence that animals could acquire this ability suggests that they could possess some of these competences, even if they may not realize any of them in their communication systems. Through the test case of negation, we investigated the phylogenetic origin of this compositional ability. We showed that baboons are able to learn an operation that can be compositionally applied to previously learned representations. Baboons thus are equipped with a rudimentary ability for skills which are often considered the hallmark of human logic and language: the manipulation of abstract representations and the ability to do so in a compositional manner.
